# Individualization of implantable devices in Brazil

**DOI:** 10.1590/0034-7167.2025780201

**Published:** 2025-02-03

**Authors:** Camila Quartim de Moraes Bruna

**Affiliations:** I Universidade de São Paulo, Escola de Enfermagem. São Paulo, São Paulo, Brazil.

The individualization of implantable devices in Brazil needs to be discussed in light of the evidence of contamination of these materials. Sterile Processment Departments (SPD), that are only managed by nurses in Brazil, need to understand the extent and severity of the issue in order to take a stand for patient safety.

An orthopedic implant is any implantable medical device indicated for use in orthopedics, with the aim of treating or correcting deformities, diseases, or injuries of the skeletal system, its joints, or associated structures. It is used in most orthopedic surgeries and is differentiated in Brazilian legislation between orthopedic implants for bone synthesis and those that are permanent.

Permanent implants are those intended for the definitive replacement of a part or function of normal body structures that have no indication for removal. Implants for bone synthesis are those intended to consolidate bone fractures in general and correct bone conformations for which removal after treatment generates much debate^(1)^.These implants are only removed in the event of infection or when they make the patient uncomfortable, and therefore become permanent.

For health standards purposes, all orthopedic implants fall into risk class III or IV, depending on the composition of the raw material. Metal implants fall into class III, while those of animal origin and those impregnated with a therapeutic or absorbable substance are risk class IV.

In addition to the categorization of health risks, legislation currently differentiates implants in terms of their commercial presentation. Permanent implants must be marketed with at least three traceability labels, whereas plates and screws, for example, do not have this requirement and have been marketed in general, in racks, in a non-individualized way.

Because they are stored in racks and trays alongside the instruments needed for their use, rather than in disposable, individual packaging, these materials circulate widely throughout hospitals and are repeatedly subjected to reprocessing. Although this is not expected, they are often handled during surgery, coming into contact with organic matter, but are not inserted into the patient, so they are returned to the rack or tray and considered “unused.”

It is not uncommon for SPD to receive these materials from outsourced companies soiled with material such as blood when they should be clean. In addition to visible soiling, viable bacteria^(2)^, corrosion, and residues of carbohydrates, fat, and detergent^(3)^ have already been identified on screws ready to be used on patients. Extensive biofilms have also been observed on screws, regardless of how long they have been on the rack.

Biofilms are related to approximately 70% of implant-related infections. An example is “aseptic releases,” which are inflammations without the identification of microorganisms that do not allow implant osseointegration, and have long been linked to biofilms. Furthermore, animal models have shown implant loss in the presence of organic residues and endotoxins, which can be deposited on implants during reprocessing.

Implants are foreign bodies that are considered sterile and are in direct and prolonged contact with body tissues, and the mere presence of these materials is considered a risk, requiring surveillance with regard to the presence of infection. Furthermore, infection is the main complication observed in implant surgeries^(4)^, and can lead to catastrophic results, which is why the sterility of these materials must be unquestionable.

Considering the risk of frequent circulation and repeated reprocessing of plates and screws, many countries have made it compulsory for these materials to be sold in disposable individual packaging. In Brazil, the sustainability of this change is still being debated, often referring back to the discussions held at the time of the replacement of glass syringes with disposable plastic syringes, a change that now seems to be unquestionable.

The advantages and disadvantages of individualizing implants in packages, such as increased surgical time and external contamination, should be discussed. Similarly, issues related to sustainability, especially in relation to excessive waste generation, should also be considered.

One consistent finding in the literature is the risk related to circulating implants as if they were ordinary surgical instruments. Nurses at the head of SPD in Brazil need to take a stand and lead the discussion on patient safety in order to tackle the commercial difficulties of migrating the presentation of these implants, including suggesting sustainably attractive options.
