# Supplementary material for: Individualization of implantable devices in Brazil
Source: Rev Bras Enferm. 2025 Feb 3;78(2):e780201. doi: 10.1590/0034-7167.2025780201 (PMC11793646; doi:10.1590/0034-7167.2025780201)
Supplement: Translation

# Translation: Individualización de dispositivos implantables en Brasil

**DOI:** 10.1590/0034-7167.2025780201es

**Authors:** Camila Quartim de Moraes Bruna

**Affiliations:** 1 Universidade de São Paulo, Escola de Enfermagem. São Paulo, São Paulo, Brasil.

La individualización de dispositivos implantables en Brasil precisa ser discutida a la luz de las evidencias de contaminación de dichos materiales. Las Centrales de Esterilización (CE), sector que en Brasil solo gestionan los enfermeras, necesitan comprender la amplitud y gravedad del tema para posicionarse en favor de la seguridad de los pacientes.

Un implante ortopédico es cualquier dispositivo médico implantable indicado para su uso en ortopedia con el fin de tratar o corregir deformidades, enfermedades o lesiones del sistema esquelético, sus articulaciones o estructuras asociadas. Son utilizados en gran parte de las cirugías ortopédicas y están diferenciados en la legislación brasileña entre implantes ortopédicos de síntesis ósea e implantes permanentes.

Los implantes permanentes son aquellos destinados a la sustitución definitiva de una parte o función correspondiente a estructuras corporales normales, que no tienen indicación de remoción. Por otro lado, los implantes de síntesis ósea son los que se utilizan para consolidar fracturas óseas en general y corregir conformaciones óseas, cuya extracción tras el tratamiento genera mucho debate.^(1)^ En la práctica, los implantes de síntesis solo se retiran por infección o cuando molestan al paciente, por lo cual acaban siendo permanentes.

A fines de clasificación sanitaria, todos los implantes ortopédicos pertenecen a la clase de riesgo III o IV, dependiendo de la composición de su materia prima. Los implantes metálicos deben clasificarse en la clase III, mientras que los de origen animal y los impregnados de sustancias terapéuticas o absorbibles tienen riesgo de clase IV.

No solo en la categorización del riesgo sanitario, la legislación diferencia hoy los implantes en su forma de presentación comercial. Los implantes permanentes deben comercializarse con al menos tres etiquetas de trazabilidad, mientras que las placas y tornillos, por ejemplo, no tienen este requisito y se han comercializado en general en racks, de manera no individualizada.

Al estar embalados en racks y bandejas junto con el instrumental necesario para su uso, en vez de en embalajes individuals descartables, estos materiales tienen una amplia circulación por los hospitales y son sometidos repetidamente a procesamiento. Aunque no sea lo esperado, con frecuencia se los manipula durante la cirugía, entrando en contacto con materia orgánica, pero no se los implanta en el paciente, volviendo así al rack o bandeja y considerándose “no utilizados".

No es extraño que los CE reciban estos materiales de empresas tercerizadas mostrando impurezas, como sangre, cuando deberían estar limpios. No solo se ha detectado suciedad visible, sino también bacterias viables,^(2)^ corrosión y residuos de carbohidratos, grasa y detergente^(3)^ en tornillos listos para ser utilizados en pacientes. Adernás, también se han observado extensos biofims en tornillos, independientemente del tiempo que hayan estado en el rack.

Los biopfilms están relacionados con aproximadamente el 70% de las infecciones relacionadas con implantes. lncluso el “aflojamiento aséptico”, inflamaciones sin identificación de microorganismos que no permite la osteointegración del implante, ha sido relacionado desde hace tiempo con los biofilms. Adernás, los modelos animales han demostrado la pérdida de implantes en presencia de resíduos orgánicos y endotoxinas, que pueden depositarse en los implantes durante su procesamiento.

Los implantes son considerados cuerpos extraños en contacto directo y prolongado con tejidos corporales considerados estériles, y la mera presencia de estos materiales está considerada un riesgo, mereciendo vigilancia en cuanto a la presencia de infecciones. Adernás, la infección es la mayor complicación observada en las cirugías con implantes,^(4)^ pudiendo provocar resultados catastróficos, por lo cual la esterilidad de estos materiales debe serincuestionable.

Considerando el riesgo de la circulación constante y del procesamiento repetido de placas y tornillos, los países han establecido la obligatoriedad de que estos materiales se vendan en embalajes individuales descartables. En Brasil, la sostenibilidad de esta migración aún está siendo debatida, remontándonos a las discusiones mantenidas en la época de la sustitución de las jeringas de vidrio por las de plástico desechable, un cambio que hoy parece ser incuestionable.

Las ventajas e desventajas de individualizar los implantes en embalajes, como el aumento del tiempo quirúrgico y la contaminación externa, merecen ser discutidos. Del mismo modo, también deberían considerarse las cuestiones relacionadas con la sostenibilidad, particularmente en relación a la generación excesiva de resíduos.

Lo que parece consistente en la literatura es el riesgo relacionado a la circulación de implantes como si fueran instrumental quirúrgico corriente. Los enfermeras al frente de los CE en Brasil necesitan posicionarse y liderar la discusión sobre la seguridad dei paciente para enfrentar las dificultades comerciales de la migración de la presentación de estos implantes, incluso sugiriendo opciones de interés a nivel de la sostenibilidad.

# Translation: Individualização de implantáveis no Brasil

**DOI:** 10.1590/0034-7167.2025780201pt

**Authors:** Camila Quartim de Moraes Bruna

**Affiliations:** 1 Universidade de São Paulo, Escola de Enfermagem. São Paulo, São Paulo, Brasil.

A individualização de implantáveis no Brasil precisa ser discutida à luz das evidências de contaminação desses materiais. Os Centros de Material e Esterilização (CME), setor que apenas a enfermagem gere no Brasil, precisam compreender a amplitude e gravidade do tema, para posicionar-se, visando à segurança dos pacientes.

Implante ortopédico é todo dispositivo médico implantável indicado para uso em ortopedia, com a finalidade de tratar ou corrigir deformidades, doenças ou lesões do sistema esquelético, suas articulações ou estruturas associadas. É utilizado em grande parte das cirurgias ortopédicas e diferenciado na legislação brasileira entre os implantes ortopédicos para síntese óssea e aqueles permanentes.

Implantes permanentes são aqueles destinados à substituição definitiva de parte ou função das estruturas normais do corpo que não têm indicação de retirada. Por outro lado, classificam-se, como implantes para síntese óssea, aqueles que se destinam à consolidação de fraturas ósseas em geral e correções de conformações ósseas, para os quais a retirada após tratamento gera bastante debate^(1)^. Na prática, implantes de síntese são retirados apenas por infecção ou quando incomodam o paciente, tornando-se, portanto, permanentes.

Para fins de enquadramento sanitário, todos os implantes ortopédicos se enquadram nas classes de risco III ou IV, dependendo da constituição da matéria prima. Implantes metálicos devem se enquadrar na classe III, enquanto os de origem animal e aqueles que estejam impregnados com substância terapêutica ou absorvível têm risco de classe IV.

Não apenas na categorização do risco sanitário, a legislação hoje diferencia os implantes na forma de apresentação comercial. Implantes permanentes devem ser comercializados com, no mínimo, três etiquetas de rastreabilidade, enquanto placas e parafusos, por exemplo, não têm essa exigência e têm sido comercializados de maneira geral, em *racks,* de maneira não individualizada.

Por serem acondicionados em *racks* e bandejas juntos com instrumental necessário à sua utilização, e não em embalagens descartáveis e individuais, esses materiais circulam amplamente pelos hospitais e são submetidos repetidas vezes ao processamento. Embora não seja o esperado, muitas vezes são manipulados durante a cirurgia, entrando em contato com matéria orgânica e, quando não inseridos no paciente, voltam, assim, ao *rack* ou bandeja, e são considerados “não utilizados”.

Não raro, nos Centros de Material e Esterilização (CME), esses materiais são recebidos das empresas terceirizadas apresentando sujidade, como sangue, quando deveriam estar limpos. Não apenas sujidade visível, mas também bactérias viáveis^(2)^, corrosão e resíduos de carboidratos, gordura e detergente^(3)^ já foram identificados em parafusos prontos para serem utilizados em pacientes. Além disso, biofilmes extensos também já foram observados em parafusos, independentemente do tempo que se encontravam à disposição no *rack.*

Biofilmes estão relacionados a aproximadamente 70% das infecções relacionadas a implantes. Mesmo as “solturas assépticas”, que são inflamações sem identificação de microrganismos que não permitem a osteointegração do implante, há muito já foram relacionadas a biofilmes. Outrossim, modelos animais já mostraram a perda do implante na presença de resíduos orgânicos e endotoxinas que podem ser depositados nos implantes durante o processamento.

Implantes são considerados corpos estranhos em contato direto e prolongado com tecidos do corpo considerados estéreis, e a simples presença desses materiais é considerada um risco, merecendo vigilância quanto à presença de infecções. Ademais, a infecção é a maior complicação observada nas cirurgias com implantes^(4)^, podendo cursar com resultados catastróficos, e, por isso, a esterilidade desses materiais deve ser inquestionável.

Considerando o risco da circulação infrene e o processamento repetido de placas e parafusos, países têm instituído a obrigatoriedade de esses materiais serem comercializados em embalagens individuais descartáveis. No Brasil, ainda se discute a sustentabilidade de realizar essa migração, remontando às discussões realizadas na época da substituição das seringas de vidro pelas seringas plásticas descartáveis, mudança que hoje parece ser inquestionável.

Discussões relacionadas a vantagens e desvantagens, como aumento do tempo cirúrgico e contaminação externa, merecem discussão. Da mesma maneira, questões relacionadas à sustentabilidade, principalmente relacionada à geração excessiva de resíduos também devem ser consideradas.

O que parece consistente na literatura é o risco relacionado à circulação de implantes como se fossem instrumental cirúrgico comum. Enfermeiros à frente dos CME no Brasil precisam se posicionar e encabeçar a discussão relativa à segurança do paciente, para enfrentar as dificuldades comerciais da migração da apresentação desses implantes, sugerindo, inclusive, opções sustentavelmente interessantes.
